# The Relationship between Iron Deficiency and Febrile Convulsion: A Case-Control Study

**DOI:** 10.5539/gjhs.v8n2p185

**Published:** 2015-06-25

**Authors:** Mohammad Reza Sharif, Davood Kheirkhah, Mahla Madani, Hamed Haddad Kashani

**Affiliations:** 1 Department of Pediatrics, Kashan University of Medical Sciences, Kashan, Iran; 2 Student Research Committee, Kashan University of Medical Sciences, Kashan, Iran; 3 Anatomical Sciences Research Center, Kashan University of Medical Sciences, Kashan, Iran

**Keywords:** iron deficiency anemia, iron deficiency, fever, febrile seizure, children

## Abstract

**Introduction:**

Febrile seizure is among the most common convulsion disorders in children, which strikes 2% to 5% of children between 3 to 60 months of age. Some studies have reported that iron deficiency could be a risk factor for febrile seizure. The present study was conducted to compare the rate of iron deficiency anemia in febrile children with and without seizure.

**Materials and Methods:**

This case-control study evaluated 200 children aged 6-60 month in two 100 person groups (febrile seizure and febrile without convulsion) in Kashan. The CBC diff, serum iron and TIBC were done for all of participants. Diagnosis of iron deficiency anemia based on mentioned tests.

**Results:**

No significant differences were found in two groups regarding to the age, gender, and the disease causing the fever. The presence of iron deficiency anemia was 45% in the convulsion group and 22% in the group with fever without convulsion. The Chi Square test indicated a significant difference between two groups.

**Conclusions:**

The findings suggest that a considerable percentage of children having febrile seizure suffer from iron-deficiency anemia and low serum iron. This means the low serum iron and presence of anemia can serve as a reinforcing factor for the febrile seizure in children.

## 1. Introduction

Febrile seizure is the most common convulsive disorder in children which strikes 2% to 5% of children between 3 to 60 months of age. Some of the recent studies have reported that iron deficiency could be a risk factor for febrile seizure because the latter is more common in children under two years and iron deficiency anemia is also common in children of the same age. Due to the presence of iron in the hemoglobin structure, it plays a crucial role in the transport of oxygen to different tissues such as the brain (Østergaard 2009; Jones et al., 2007; Kheirkhah et al., 2013; Kliegman 2011; Pisacane et al., 1996). Iron deficiency reduces the metabolism of some neurotransmitters (Lozoff et al., 2006; Parks et al., 1989). Several lines of evidence led to the hypothesis that iron deficiency can have a role in the onset of a convulsion. However, the studies carried out so far have reported conflicting results. Some studies have reported that in the patients with iron deficiency, febrile convulsion is significantly higher than that in the control group (Ur-Rehman et al., 2005; Daoud et al., 2002; Hartfield et al., 2009; Momen et al., 2010). On the contrary, some authors have concluded that the risk of febrile seizure in anemic children seems to be less than that in children with no febrile seizure (Talebian et al., 2006) and that iron deficiency can be a protective mechanism against convulsions by increasing the convulsion threshold (Kobrinsky et al., 1995). Other studies have shown that iron deficiency plays no role in pediatric febrile seizures (Salehi et al., 2009; Amirsalari et al., 2010). Since the relationship between iron deficiency and febrile seizure is not yet determined, chance or other unknown factors can be considered as causes (Bidabadi et al., 2009). Considering the above results and since no study has been conducted in Kashan, Iran on the mentioned relationship, the present study was conducted to compare the iron deficiency anemia in the febrile seizure children to those in the febrile patients without convulsion.

## 2. Methods and Materials

This is a case-control study, which was conducted during 2012 in Kashan, on 200 participants in two 100-person groups: febrile seizure and fever without convulsion. The case group consisted of children, aged 6 months to 5 years, with normal development, who were taken to the hospital for the first febrile seizure. Samples with electrolyte imbalance (sodium, calcium, etc.), hypoglycemia, meningitis, encephalitis, shigellosis, neurological deficiencies and history of seizure (with or without fever) were excluded from the study. For each child with febrile convulsion, a child aged 6-60 months was selected in the control group with a similar febrile illness, but didn’t show seizure. In all cases, after the goals and the voluntary nature of the study were explained to the parents, they were asked to sign the consent form. Personal information about the children, including age, gender and family history of seizure were collected from parents through an interviewed questionnaire. In both groups, body temperature was also measured and recorded by axillary method. The blood tests of CBC diff, serum Iron, and TIBC were conducted. Anemia was defined as Hb < 10.5g/dl for children from the age of six months to two year olds, and Hb < 11.5g/dl for 2-5 years old children. The normal level of serum iron was determined as Fe > 40 µg/dl for children younger than one year and Fe > 50 for children over one year. The normal range of TIBC was considered 210-430 µg/dl. The normal transferrin saturation percentage was considered higher than 15 percent (Kliegman, 2011; Oski et al., 2008). Diagnosis of iron deficiency anemia based on mentioned criteria. The collected data were analyzed using descriptive statistics, including frequency, percentage, standard deviation and mean, as well as analytical statistics, such as Chi Square to compare qualitative variables; the Analysis of variance test and T-test to compare quantitative variables.

## 3. Results

When studying the personal characteristics, no significant differences were found in two groups in terms of age, gender. Regarding the family history of convulsion, there was a significant difference between the study groups. Furthermore, the febrile convulsion group and the group of fever without convulsion had no significant difference in terms of the disease causing the fever. The diseases causing the fever in both groups were respiratory and digestive infections (Table 1).

**Table 1:** Personal information of patients under study in two groups

Personal information	Group	Febrile seizure n(%)	Fever without convulsion n(%)	PV
**Gender**	**Boy**	62(62)	56(56)	0.38
	**Girl**	38(38)	44(44)	
**Family history of seizure**	**Yes**	27(27)	10(10.1)	0.002
	**No**	73(73)	89(89.9)	
**Infectious disease type**	**Respiratory**	64(64)	60(60)	0.49
	**Gastroenteritis**	33(33)	38(38)	
	**Others**	3(3)	2(2)	
Age (month) *		25.34±12.56	24.78±14.56	0.77

* Mean ± standard deviation.

In the febrile convulsion group, most of the members (72%) belonged to the age group of less than two years (18% were less than one year, 36% between 1 and 2 years old, 25% between two and three, and 21% over three years). The average of serum Iron and TIBC were significantly different in two groups (Table 2).

**Table 2:** Mean and standard deviation of different indices of iron deficiency anemia in children under study

Group iron anemia	Febrile Seizure	Fever without convulsion	PV
**Hb**	11.45±1.34	11.82±1.45	0.062
**Serum iron**	42.62 ±28.02	52.45±34.76	0.028
**TIBC**	393.22±39.61	345.94±26.82	0.0001
**Transferrin saturation percentage**	12.77 ±8.26	14.41±10.60	0.22

The presence of iron deficiency anemia was 45% in the convulsion group, 22% in the group with fever without convulsion. The Chi Square test indicated a significant difference between the groups (Table 3).

**Table 3:** Frequency distribution of iron deficiency anemia in the children of two groups

Iron deficiency anemia Group	Positive n(%)	Negative n(%)	PV
**Febrile Seizure**	45(67.1)	55(41.35)	0.0005
**Fever without convulsion**	22(32.9)	78(58.65)	
**Total**	67(100)	133(100)	

## 4. Discussion

In this study, the incidence of iron-deficiency anemia in the febrile convulsion group was obviously higher than the control group. Like our research, the study of Pisacane et al reported that anemia in the case group (30%) was higher than in the hospital control group (14%) and the healthy group (12%) (Pisacane et al., 1996). In the study of Vaswani et al also, 68% of cases were iron deficient compared to 30% of the controls (Vaswani et al., 2009). In the study of Sadeghzadeh et al, although anemia was not common among febrile seizure patients, iron deficiency was more frequent in these patients (Sadeghzadeh et al., 2012). A study by Ur-Rahman and Billoo on 30 children with febrile convulsion and 30 children with other febrile diseases indicated that iron-deficiency anemia in the case group was significantly more common than in the control group (Ur-Rehman et al., 2005). A Kenyan case-control study as well as the meta-analysis of eight case-control studies that have examined the relationship between febrile seizures or acute seizures and iron deficiency suggested that iron deficiency may be associated with an increased risk of febrile seizures in children (Idro et al., 2010). Fever can worsen the effects of anemia or iron deficiency on the brain, and therefore cause convulsions. In addition, anemia can be associated with the degree of febrile disease, and patients with more severe symptoms may be affected by convulsions. But, febrile convulsion usually occurs at the onset of a febrile disease, before hemoglobin is reduced due to the infectious disease (Pisacane et al., 1996). In a study conducted in Thailand, the rate of thalassemic children with febrile convulsion was reported as being 4.4 times less than the general population of children. The researchers suggested that it might be due to higher levels and the role of iron in brain metabolism, which leads to less occurrence of febrile convulsion in those children (Auvichayapat et al., 2004). This study, of course, can simply assess its role in increasing the iron and in the reduction of febrile convulsions, and cannot be a desirable scale to measure iron-deficiency anemia and febrile convulsion. On the other hand, low risk of febrile convulsion in thalassemic patients could be due to several other clinical conditions that they may have. On the other hand, some studies have reported findings that are not similar to the result of this study. For example, in the study of Hartfield et al, iron deficiency was found to be 9% and 5% in the children of two groups of febrile convulsion and febrile without convulsion, respectively; and iron-deficiency anemia was found to be 6% and 4% in the former and latter groups, respectively (Hartfield et al., 2009). Again, in the study of Kobrinsky et al, the febrile convulsion group suffered less from iron deficiency. They concluded that iron deficiency could have a protective effect against febrile convulsions (Hartfield et al., 2009); and in the paper by Bidabadi, iron deficiency in the febrile convulsion group (44%) was less than in the control group (48%), but since there was no significant difference, the protective effect of iron deficiency against febrile convulsions was not confirmed (Bidabadi et al., 2009). The major causes that lead to different results between their and our studies may include not considering the effect of age in interpreting the tests for the diagnosis of iron deficiency, the difference in the age and number of samples, and difference in the diagnostic criteria of IDA between their and our studies. In the present study, all samples of the case group suffered from the febrile convulsion for the first time, but in most of the mentioned studies, some samples had a history of febrile convulsion.

According the findings of the present study, the incidence of iron deficiency in children suffering from fever and convulsion was observed to be more than that the fever without convulsion group. In conclusion, the findings suggest that low serum iron levels and the presence of anemia can serve as strengthening factors for the febrile seizures in children. Thus, iron deficiency can be added to the list of risk factors for febrile convulsions. Accordingly, children with febrile seizures are suggested to be monitored for diagnosis and treatment of iron-deficiency anemia. Furthermore, it is advisable to prescribe the iron supplements sooner and more carefully to children who have important and well-known risk factors for febrile convulsion, such as family history of febrile convulsion. It will be worthwhile to conduct a study to follow up children with iron deficiency, which stricken by the febrile convulsions after the treatment of iron deficiency, in terms of the recurrence rate of febrile convulsions.

## 5. Conclusions

The findings suggest that a considerable percentage of children having febrile seizure suffer from iron-deficiency anemia and low serum iron. This means the low serum iron and presence of anemia can serve as a reinforcing factor for the febrile seizure in children.
